# Research highlights for issue 2: recent applications in molecular evolution

**DOI:** 10.1111/eva.12246

**Published:** 2015-01-28

**Authors:** Britt Koskella

**Affiliations:** Research Highlights Associate Editor Evolutionary Applications

The study of changing sequence composition of DNA, RNA and proteins over time has offered some of the most fundamental insights into the evolutionary process to date. From understanding how populations and ultimately species diverge to the study of how particular selection pressures affect changes in genotype and phenotype, our knowledge of evolution would be a fraction of what it is now without the major advances made in the field of molecular evolution. Recent technological and bioinformatical improvements have continued to expand these insights, and have also offered key applications such as the ability to model and predict pathogen evolution, monitor the effective population size of threatened species, and help understand what constitutes a healthy microbiome.

Two recent studies, both led by Nora Besansky and published in *Science,* emphasize the power and challenges of comparative genomics when working to understand the evolution of disease vectors. First, Daniel Neafsey and colleagues report the sequencing, assembly, and comparison of genomes from 16 Anopheles mosquito species (Neafsey et al. 2014). As 11 of these species are considered major disease vectors, comparison among the genomes allowed the researchers to examine underlying genes that may be associated with vectoring capacity. The results suggest that, relative to the Drosophila genus, the Anopholes' genomes are remarkably flexible, with rapid rates of gene loss/gain, increased loss of introns, and shuffling of genes on the X chromosome. The data suggest a mechanism for the observed functional diversity across the species, especially in those traits such as chemosensory ability that are associated with adaptation to host feeding and therefore disease vectoring. However, comparison among genomes was hampered by what are most likely high levels of interspecific gene flow, or introgression, as described in a separate paper by Michael Fontaine and coauthors (Fontaine et al. 2014). Depending on which genomic segment the authors used to build phylogenetic trees, a remarkably different pattern emerged; trees based on autosomal sequences tended to group the three major vectors of malaria together, while those built using the X chromosome suggest early radiation of these three species and persistent introgression on the autosomes. Together, these studies offer tantalizing hypotheses for the adaptive significance of among-species gene flow and genomic plasticity in allowing the Anopholes genus to act as vectors for a wide array of pathogens.

In addition to the increasing power of genomics and phylogenomics, the use of transcriptional profiling has also proven invaluable to the field. A recent review of novel insights gained through transcriptomic analyses of natural populations by Mariano Alvarez and collaborators highlights the utility of this approach in testing how genotype translates to phenotype, and how this translation is influenced by environment-specific gene expression (Alvarez et al. 2014). Such variation can have dramatic implications for the process of adaptation as well as our ability to predict the response of populations to rapid environmental changes such as those resulting from pathogens, pollutants, or climate change. More recent advancement in transcriptomics includes the ability to profile gene expression of single cells, as discussed by Nicola Crosetto and coauthors in a new paper reviewing recent progress in spatiotemporal transcriptomics (Crosetto et al. 2015). Among the many applications of this powerful approach to unravelling among-cell expression differences is the ability to examine heterogeneity of tumour cells to predict drug sensitivity of various cancers.

The use of sequence data to infer evolutionary processes is not limited to single species. Indeed, the use of metagenomics to infer the composition of species from environmental samples has greatly enhanced our understanding of microbial diversity. In its simplest form, metagenomic analysis allows for a culture-independent characterization of microbial community composition. This type of analysis has gained much recent attention for its application in understanding the microbiomes of eukaryotic species. For example, recent work by Julia Goodrich and colleagues examined how human genetics shapes the relative abundances of various gut bacteria by comparing microbiotas across 416 pairs of twins (Goodrich et al. 2014). The authors first discovered a clear heritability for a subset of bacterial taxa, most notably those from the family Christensenellaceae, which were also correlated with low host body-mass index (BMI). The authors then went a step further by adding a particular species of Christensenellaceae into an obese-associated microbiome and inoculating sterile mice with either the unaltered or altered microbial community. In this way, they were able to demonstrate not only correlation with host metabolism in humans but also to infer causation, as mice supplemented with this species showed reduced weight gain relative to those not receiving the supplement.

The simultaneous analysis of multiple genomes within a single environmental sample also allows for assessment of selection acting on genes shared by members of the community. A terrific example of this comes from recent work by Molly Gibson and collaborators who examined the so-called ‘resistome’ of microbial communities from soil and the human gut, in this case focusing on the genes conferring resistance against 18 antibiotics typically used in clinical settings (Gibson et al. 2015). The authors used a new database of protein families to assign antibiotic resistance functions to each metagenomic segment, and were able to demonstrate that the antibiotic resistance genes found in environmental versus human-associated microbiota were functionally different, perhaps suggesting less gene flow among these communities than previously thought.

Overall, the recent advancements in both omics and bioinformatics have been game-changing for the field of molecular evolution, and the application of such new approaches and technologies have only begun to surface. The potential for advancement in clinical and agricultural settings is already being realized, and application to the management of natural populations, including the spread of disease, is already following.
